# Different clinical course of BPPV according to the medical conditions

**DOI:** 10.1038/s41598-024-63882-3

**Published:** 2024-06-05

**Authors:** Jeon Mi Lee, Hyun Jin Lee

**Affiliations:** 1 Department of Otorhinolaryngology, Ilsan Paik Hospital, Inje University College of Medicine, Goyang, Korea; 2 Department of Otorhinolaryngology-Head and Neck Surgery, Incheon St. Mary’s Hospital, College of Medicine, The Catholic University of Korea, Seoul, Korea

**Keywords:** BPPV, Trauma, Hospitalization, Neurological disorders, Neurological disorders

## Abstract

Dizziness is one of the most prevalent complaints in medicine, and benign paroxysmal positional vertigo (BPPV) accounts for one-third of all cases. The present study was aimed at identifying differences in the course and prognosis of BPPV depending on the patient’s medical condition during hospitalization. Patients in group 1 were hospitalized due to trauma, those in group 2 for scheduled surgery, and those in group 3 for medical treatment. The intervals from admission to symptom onset, surgery to symptom onset, and symptom onset to ENT department referral were compared. The interval from admission to symptom onset was shortest in group 1 (3.1 ± 8.0 days) and differed significantly from that in group 3 (20.0 ± 35.0 days, *p* < 0.001). The interval from surgery to symptom onset for group 2 was 5.6 ± 5.8 days and was significantly shorter than that from admission to symptom onset for group 3 (*p* = 0.014). The interval from symptom onset to ENT referral in group 3 (2.0 ± 2.8 days) was significantly shorter than in groups 1 and 2 (4.1 ± 5.1 and 4.0 ± 3.6 days, *p* = 0.008 and *p* = 0.002, respectively). The findings imply that the course of BPPV differed according to the patients’ medical condition.

## Introduction

Dizziness is one of the most prevalent complaints in medicine, affecting 15–35% of the general population^1^, and benign paroxysmal positional vertigo (BPPV) accounts for approximately one-third of all cases^2^. BPPV is the most common peripheral vestibular disorder, with a lifetime prevalence of 2.4% and a 1-year incidence rate of 0.6%^2^. Previous researchers have proposed the pathophysiological concept of BPPV as follows: otolithic debris from the utricular macule migrates into the semicircular canal, causing vertigo and nystagmus by inducing endolymph flow during positional changes^3^. In 50–70% of cases, the cause of otolith detachment is unknown (idiopathic)^4^, while hormonal factors and calcium metabolism are thought to play important roles in the development of BPPV in view of its high prevalence in middle-aged women^5^. Secondary BPPV refers to a condition in which the otoliths detach from the utricle owing to an underlying pathology. Head trauma, migraine, and inner ear diseases such as vestibular neuritis, Meniere’s disease, and labyrinthitis can lead to secondary BPPV. The main symptom of BPPV is vertigo, which is induced by changes in the head position. It is mostly transient and usually resolves within 1 minute^6^. However, symptoms may differ among patients and include nonspecific dizziness, lightheadedness, postural instability, persistent dizziness, and imbalance. In some cases, the symptoms may be mild and do not require a hospital visit, but in others, patients may experience severe dizziness and require a visit to the emergency room. According to a recent report, 9.8% of patients who visited the emergency room with dizziness had BPPV^7^.

This applies not only to the general population but also to people who are hospitalized for reasons other than dizziness. Dizziness can occur while staying in the hospital, and some patients might have BPPV. However, when hospitalized patients newly develop dizziness, BPPV is not easily suspected. Dizziness can result from drug side effects or surgery under general anesthesia. Furthermore, there are numerous cases of nonspecific dizziness due to deterioration in individuals’ general condition. Still, it is clear that BPPV also accounts for a portion of dizziness cases. There have been some reports of patients who developed BPPV during hospitalization. However, most are case reports, and no large-scale studies on the topic have been published^8–10^.

Because BPPV is not easily suspected when a hospitalized patient complains of dizziness, it is important to determine which patients develop BPPV during hospitalization and whether the nature of BPPV or treatment effects differ depending on the patient group. This information may facilitate the early determination and proper treatment of BPPV among patients who newly develop dizziness during hospitalization. The present study was aimed at identifying differences in the course and prognosis of BPPV depending on the patient’s medical condition and analyzing the clinical characteristics of BPPV during hospitalization.

## Methods

### Participants

Patients who developed BPPV and were treated between January 2020 and June 2023 at two distinct hospitals were retrospectively evaluated. The patients were hospitalized for various reasons and classified into three groups. Group 1 included patients who were hospitalized owing to head trauma. Group 2 included patients who were hospitalized for a scheduled surgery, excluding head and neck surgery, since head and neck surgery can be another cause of secondary BPPV^11^. Finally, group 3 included patients who were hospitalized for medical treatments such as cardiovascular disease, infection, anticancer, or psychiatric treatment. Those who were hospitalized because of dizziness, complained of dizziness before hospitalization, or were unconscious at the time of hospitalization were excluded. Data on the presence of hypertension and diabetes were also collected.

### Diagnosis and treatment

The patient was referred to the otolaryngology (ENT) department because of dizziness during hospitalization. Patients were examined by ENT specialists and diagnosed with BPPV according to the diagnostic criteria provided by the Bárány Society^12^. After the diagnosis of BPPV, all patients underwent a canalith repositioning maneuver (CRM) according to the BPPV subtype. The treatment procedures for each BPPV subtype were the modified Epley maneuver for posterior semicircular canal BPPV (PC-BPPV), modified Lempert maneuver for geotropic-type horizontal semicircular canal BPPV (HC-BPPV), and cupulolith repositioning maneuver for apogeotropic-type HC-BPPV^13^. All patients underwent positional tests after 1 week for reassessment and received a single CRM until resolution. If the patient did not show positional vertigo or nystagmus during follow-up, the condition was considered to have been resolved. If the patients were discharged before resolution, they were asked to visit the ENT outpatient department and the same procedure was performed. The following variables were collected and compared: the interval from admission to symptom onset, surgery to symptom onset, and symptom onset to referral to the ENT department. Additionally, the subtypes of BPPV and the number of CRMs required to achieve resolution were compared.

### Statistical analysis

All statistical analyses were performed using GraphPad Prism 8 for Windows (GraphPad Software, La Jolla, CA, USA) and SPSS software version 21 for Windows (IBM Corp., Armonk, NY, USA). The results of descriptive statistics are expressed as mean ± standard deviation values. The visual examination of Q-Q normality plots and Shapiro–Wilk test suggested that not all parameters satisfied normality, hence non-parametric tests were used. The variables were compared among the three groups by using Kruskal–Wallis with Bonferroni post hoc analysis and Pearson’s chi-square test. When the expected value is less than 5, Fisher’s exact test was performed. Statistical significance was set at *p* < 0.05.

### Ethical statement

This study was approved by the institutional review board (approval no. 2023–12-012 and OC23RASI0160), and the requirement for informed consent was waived. The study was performed in accordance with the tenets of the Declaration of Helsinki and Good Clinical Practice guidelines.

## Results

### Study population

**Figure 1:**
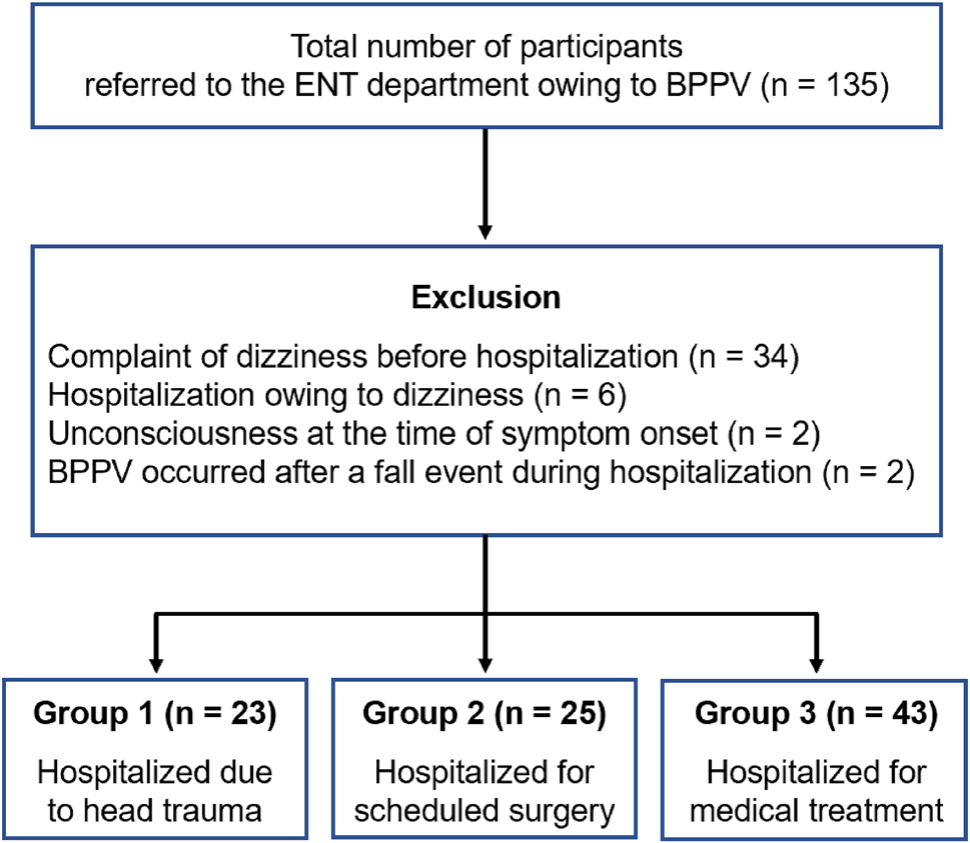
Schematic illustration of participant selection in the present study. BPPV: benign paroxysmal positional vertigo

Overall, 135 patients were referred to the ENT department because of dizziness and were diagnosed with BPPV. Thirty-four patients were excluded because they complained of dizziness before hospitalization, and six were excluded because they were hospitalized due to dizziness. Two patients were excluded because they were unconscious during hospitalization and the exact time of symptom onset could not be confirmed; another two patients were excluded because BPPV occurred after a fall event during hospitalization. Ultimately, 91 patients were included in this study. The patients were classified into three groups. Groups 1, 2, and 3 were hospitalized owing to head trauma (*n* = 23), scheduled surgery (*n* = 25), and medical treatment (*n* = 43; Fig. 1).

The average age of groups 1, 2, and 3 were 57.0 ± 17.9, 69.4 ± 9.4, and 68.4 ± 17.4 years, respectively. There were significant age differences between groups 1 and 2 (*p* = 0.004) and between groups 1 and 3 (*p* = 0.005); however, no difference was found between groups 2 and 3. Female patients outnumbered male patients by 30–68% in all groups, and the difference in proportion was not significant between the groups. The prevalence of hypertension was significantly higher in groups 2 (*p* = 0.003) and 3 (*p* = 0.031) than in group 1, whereas the prevalence of diabetes was significantly higher in group 3 than in group 1 (*p* = 0.004). When the prevalence rates of hypertension and diabetes were combined, significant differences were found between groups 1 and 2 (*p* < 0.001) and between groups 1 and 3 (*p* = 0.005). The patients’ demographic data are presented in Table 1.

**Table 1:** Demographics of the patients.

	Group 1 (*n* = 23)	Group 2 (*n* = 25)	Group 3 (*n* = 43)	*p*-value
Age, mean ± SD, *y*	57.0 ± 17.9	69.4 ± 9.4	68.4 ± 17.4	**0.006**
Sex (M:F), *n*	10:13	10:15	16:27	0.884
Hypertension (%)	4/23 (17.4)	15/25 (60.0)	19/43 (44.2)	**0.011**
Diabetes mellitus (%)	1/23 (4.3)	6/25 (24.0)	16/43 (37.2)	**0.014**
Hypertension and diabetes Mellitus (%)	4/23 (17.4)	17/25 (68.0)	23/43 (53.5)	**0.002**

Significant values are in [bold].

### Time differences in admission, symptom onset, and referral to ENT department among the groups

We compared the interval from admission to symptom onset among the groups. Group 1 showed the shortest interval of 3.1 ± 8.0 days, and groups 2 and 3 had an interval of 11.2 ± 18.1 and 20.0 ± 35.0 days, respectively. There was a statistically significant difference between groups 1 and 3 (*p* < 0.001). Groups 2 and 3 also showed a time difference of approximately 9 days; this difference was statistically insignificant. Since group 2 represented the patients who received the scheduled surgery, we also collected data for the interval from surgery to symptom onset in group 2 and compared it with the interval from admission to symptom onset in groups 1 and 3. In group 2, symptoms developed 5.6 ± 5.8 days after receiving the scheduled surgery, and this was significantly different from the interval from admission to symptom onset in group 3 (*p* = 0.014).

**Figure 2:**
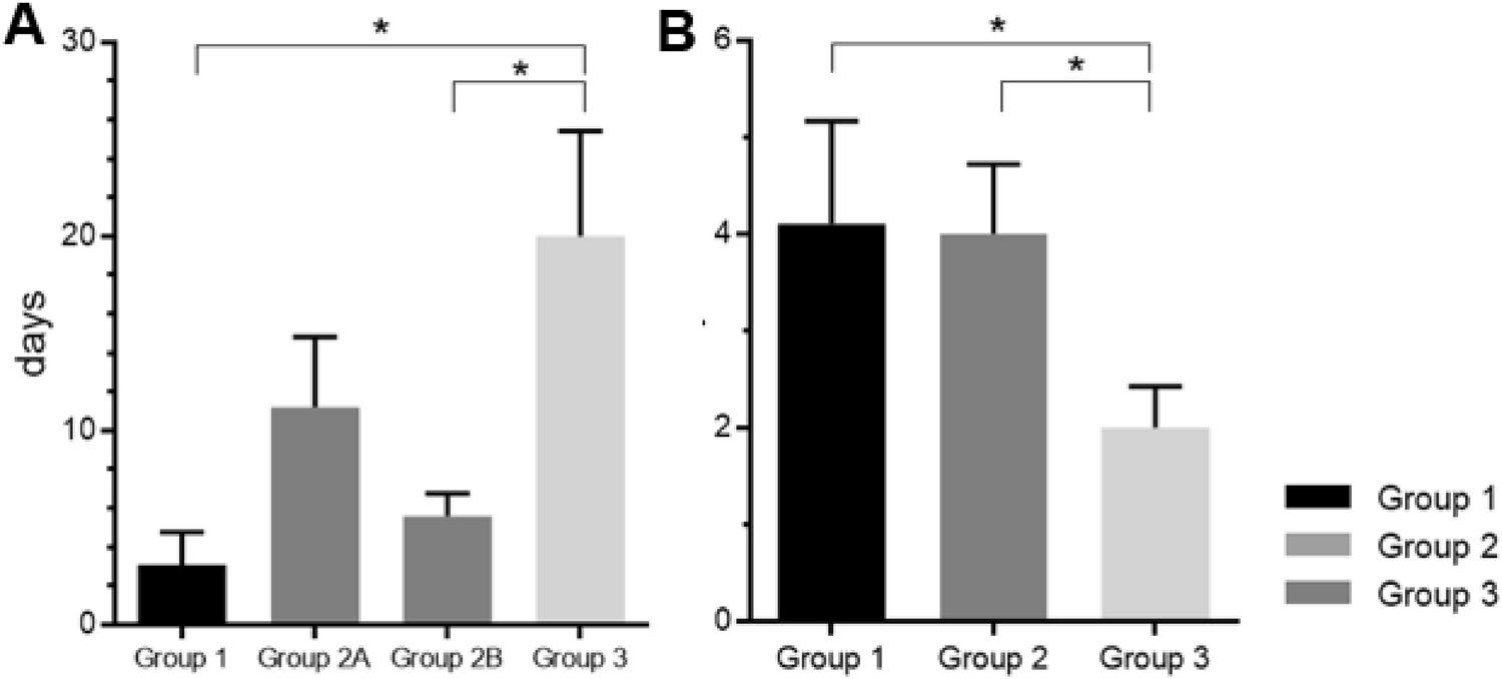
Time differences in events among the group. (**A**) Time differences from admission/surgery to symptom onset. (**B**) Time differences from symptom onset to referral to the ENT department Group 2A; Time from admission to symptom onset, Group 2B; Time from surgery to symptom onset.

The interval from symptom onset to referral to the ENT department also differed among the groups. Patients in group 3 exhibited the shortest interval (2.0 ± 2.8 days) to contact the ENT department, while groups 1 and 2 took 4.1 ± 5.1 and 4.0 ± 3.6 days, respectively (*p* = 0.008 and *p* = 0.002, respectively). Figure 2 shows the time differences in events among the groups.

### Disease characteristics among the groups

We attempted to determine the differences in the characteristics of BPPV among the groups. There was no predominant location of occurrence between the left and right sides, and there was no difference in the location of occurrence among the three groups. Although there was no difference in the canals involved among the three groups, HC-BPPV was more common in groups 1 and 2, and PC-BPPV was more common in group 3. Anterior semicircular canal BPPV (AC-BPPV) was noted in one patient each in groups 2 and 3. When AC-BPPV was excluded and only HC-BPPV and PC-BPPV were compared, there was no difference among the groups. We also compared geotropic and apogeotropic subtypes of HC-BPPV. The geotropic type was slightly more common than the apogeotropic type; however, there was no significant difference among the groups (Table 2).

**Table 2:** Characteristics of BPPV in hospitalized patients.

	Group 1 (*n* = 23)	Group 2 (*n* = 25)	Group 3 (*n* = 43)	*p*-value
Site (R:L)	13:10	11:14	20:23	0.653
Type (HC-BPPV:PC-BPPV:AC-BPPV)	12:11:0	15:9:1	17:25:1	0.282
Subtype of HC-BPPV (Canalolithiasis: Cupulolithiasis)	7:5	9:6	9:8	0.346
Number of CRMs, mean (SD)	1.6 (1.1)	1.4 (0.6)	1.4 (0.8)	0.711

The number of CRMs required to achieve reduction was determined and compared. Groups 1, 2, and 3 received 1.6 ± 1.1, 1.4 ± 0.6, and 1.4 ± 0.8 CRMs, respectively, until resolution. There was no significant difference in the number of CRMs among the groups (Table 2).

R; right, L; left, HC-BPPV: horizontal semicircular canal BPPV, PC-BPPV: posterior semicircular canal BPPV, AC-BPPV: anterior semicircular canal BPPV, CRM: canalith repositioning maneuver, BPPV: benign paroxysmal positional vertigo.

## Discussion

The main findings of this study are as follows. First, BPPV can occur in hospitalized patients for reasons unrelated to dizziness. Second, the interval to development of BPPV varies according to the cause and type of hospitalization. Third, there were no significant differences in the type of BPPV, number of CRMs required until resolution, or treatment effect, regardless of the cause and type of hospitalization.

In the present study, patients were classified into three groups. Group 1 included patients hospitalized for head trauma. Most patients had a direct injury and had the shortest time for BPPV development, with the average period being 3.1 days. This finding was significantly different from that in group 3 patients, who were hospitalized for medical treatment, which took an average of 20 days. This difference is likely due to the different mechanisms underlying the development of BPPV. Group 1 included the patients with traumatic BPPV (t-BPPV). Compared to idiopathic BPPV (i-BPPV), which develops owing to long-term otolith degeneration, a direct impact on the inner ear structure causes otolith detachment more quickly in t-BPPV. Therefore, it is reasonable to conclude that group 1 developed symptoms shortly after hospitalization. Considering the distinctive characteristics of t-BPPV compared with those of i-BPPV^14^, it is also understandable that group 1 patients were significantly younger than those in the other groups.

There was no difference in the interval from admission to symptom onset between groups 2 and 3; however, when the standard was set as the time of the scheduled surgery, a significant difference was found. The two groups were comparable in age, sex, and prevalence of hypertension and diabetes; thus, this difference should be attributable to surgery. An average interval of 5.6 days from surgery to symptom onset was comparable with that in a previous study. A case series of seven patients published by Gyo also indicated that BPPV developed in an average of 6.9 days following surgery^8^. It is unclear which surgical factors influence the development of BPPV; however, a few possibilities have been proposed. There was a suggestion that transient hypotension and hypoperfusion of the inner ear during surgery could have affected the development of BPPV^15^. Meanwhile, another study used the vestibular evoked myogenic potential test to investigate the effect of general anesthesia on the vestibular system and concluded that anesthetics had no influence on the vestibular system^16^. Furthermore, some cases of BPPV development following surgery in the Trendelenburg position have been described, and this position during surgery has been presented as a cause of postoperative BPPV^17^. However, the majority of the group 2 patients underwent back or knee surgery and received surgeries in the supine and prone positions. Gyo also suggested that prolonged bed rest after surgery can contribute to the onset of BPPV, as evidenced by cases where symptoms appeared upon initial mobilization after several days of postoperative immobility^8^. However, a noticeable difference in the interval between surgery and BPPV onset was observed in patients from group 3, who also had also been bedridden during hospitalization. Given that the majority of patients in group 2 underwent disc herniation surgery and total knee replacement arthroscopy, although this was not examined in the present study, metabolic bone diseases such as osteoporosis may also be taken into consideration. Numerous studies have revealed a connection between osteoporosis and orthopedic patients, and there is a strong correlation between osteoporosis and BPPV^18^. Non-orthopedic patients were also included in group 2, and it might be challenging to identify the precise cause.

Group 3 included patients who received chemotherapy; psychiatric patients; patients with leukopenia, angina, lung-related diseases, and gastrointestinal tract-related diseases. These patients developed symptoms in an average of 20 days. Considering that the majority of patients spent most of their hospital stay in bed, this result is comparable to the findings of previous research. Wang et al. conducted an interesting study with healthy subjects lying down for 90 days and examined whether they developed BPPV^19^. They were strictly positioned in an exact 6-degree head-down prone position for 90 days, and five out of 36 healthy subjects developed BPPV during 17 to 42 days of their bed rest. Researchers have suggested that gravity plays an important role in otolith detachment. BPPV mainly develops in the semicircular canal, which is the most affected by gravity, depending on the subject’s lying position. Lopez et al. found that subjects who mainly sleep in the left or right lateral position experienced PC-BPPV on the ipsilateral side^20^. Wang et al. found that all patients developed HC-BPPV; the canal was most affected by gravity in the 6-degree prone position^19^. Although the intergroup difference was not significant, the incidence of PC-BPPV was higher in group 3 than in groups 1 and 2, which can also be assumed to be owing to the influence of gravity. However, not everyone develops BPPV in the long-term lying down position. Because the study by Wang et al. also showed that patients who developed BPPV had significantly lower 25-hydroxy-vitamin D levels than did those who did not, BPPV would result from the interaction of gravity and a situation where several factors are present for otolith detachment.

There were differences in the time from symptom onset to referral to ENT department among the groups. It took an average of 4 days for groups 1 and 2; however, for group 3, a consultation was requested in just 2 days. Patients commonly experience dizziness after trauma or general anesthesia. Dizziness after general anesthesia is typically accompanied by nausea and lightheadedness and resolves within 2–5 days with conservative treatment^21^. In the case of post-traumatic dizziness, various manifestations ranging from central dizziness due to cerebral hemorrhage to malingering were noted^22^. It takes more time for clinicians to suspect BPPV and request consultation with the ENT department in situations where dizziness can commonly occur. Patients with BPPV may complain of extreme vertigo and an immediate CRM may be required. It is important to consider these differences and treat patients appropriately when they complain of dizziness following trauma or general anesthesia.

Although the time to symptom development and the possible etiologies were thought to differ depending on the cause and type of hospitalization, there was no significant difference in the treatment effect among the groups. Most patients recovered from BPPV after an average of 1.5 rounds of CRM, regardless of the group. Currently, there are varying opinions regarding the treatment effects in patients with t-BPPV. Most studies have argued that patients with t-BPPV require more CRMs than those with i-BPPV, and a recent meta-analysis of 860 patients with t-BPPV also supported this^14^. Another study also reported that the bilateral involvement rate in t-BPPV was 4.67-fold higher. The authors found that the persistence rate after CRM increased with the severity of trauma, but the recurrence rate was not statistically different between t-BPPV and i-BPPV. They interpreted this observation by attributing the worsened results of the treatment to the unexpected effect of trauma on the vestibular organ leading to the involvement of multiple canals, but the natural courses of t-BPPV and i-BPPV were similar^23^. Within the same context, it was reported in yet another study involving 110 patients with t-BPPV from a single institution that the effect of treatment was comparable to that in patients with i-BPPV^24^. These results have been explained by emphasizing that bilateral involvement and atypical BPPV are more commonly found in cases of t-BPPV. Taken together, these pieces of evidence suggest that complete resolution in t-BPPV might not be achieved due to underdiagnosis of the bilateral and atypical forms. In this study, all patients in group 1 presented with typical unilateral BPPV. They were also conscious at the time of injury, indicating that the head trauma could be classified as less than “severe”. This could provide a sufficient explanation for our results.

Lopez et al. emphasized that the number of agglomerated particles or size of the debris is important for otolith detachment from the utricle under the influence of gravity. It is highly likely that in t-BPPV, in which otolith detachment occurs owing to direct impact, otoliths have a sufficiently low weight to prevent them from being naturally dislodged from the utricle by gravity. In general, the smaller the otolith, the longer the time required for otoconia to move inside the semicircular canals^25^. However, if CRMs are performed properly with sufficient time between the procedures, even small otoliths can pass through the canal sufficiently well to enter the utricle. The unfavorable outcomes of t-BPPV might be owing to the small size of the otoliths, necessitating sufficient time for each CRM, which may not have been done properly. In the present study, all CRMs were performed by ENT specialists, and we performed proper CRMs, ensuring that the nystagmus disappeared after each procedure before proceeding to the next procedure, according to the protocol. We believe that these factors explain why there was no significant difference in the treatment effect among the groups.

The limitations of this study were as follows. First, we could not confirm underlying factors other than hypertension and diabetes. In particular, osteoporosis or a history of BPPV and otologic disease, which are known to be significantly associated with the development of BPPV, could not be confirmed. In addition, we re-examined the patients every week, and the long interval between examinations may not have reflected the exact treatment effects among the groups. On the other hand, considering that the average time required for the natural resolution of untreated BPPV is 16–39 days^26^, follow-up observations at 1-week intervals would not have made a very large difference. Lastly, we did not classify PC-BPPV into subtypes. PC-BPPV can present as either canalolithiasis (PC-BPPV-ca) or cupulolithiasis (PC-BPPV-cu). The Bárány Society defined PC-BPPV-cu as when positional nystagmus is elicited after a brief or no latency by the Dix-Hallpike maneuver, lasting > 1 min^12^. Since PC-BPPV-ca accounts for the majority of PC-BPPV cases, and there are no specific treatment options based on PC-BPPV subtype, both PC-BPPV-ca and PC-BPPV-cu share the same CRM (modified Epley maneuver). Thus, we did not necessarily attempt to distinguish between the two by observing nystagmus for more than 1 min. However, a previous study suggested that the two subtypes might have different pathology and treatment response, as evidenced by the fact that a CRM that was effective for PC-BPPV-ca did not have a significant effect for PC-BPPV-cu^27^. Unidentified PC-BPPV-cu could have influenced the study results. However, PC-BPPV-cu is very rare, accounting for only approximately 7.2% of all cases of PC-BPPV^28^, and therefore this is unlikely to have had a significant impact on the study results.

## Conclusion

To our knowledge, the present study is the first to analyze patients who were hospitalized for reasons other than dizziness and developed BPPV. We confirmed that the course of BPPV differed according to the patients’ medical condition, but treatment effects were comparable. Dizziness commonly occurs in hospitalized patients, and it can be easily overlooked due to common causes, such as drug side effects, general anesthesia, or trauma. However, BPPV should also be suspected as it can be easily treated if diagnosed accurately and with proper CRMs (Supplementary Table).

### Supplementary Information

Supplementary Information.

## Data Availability

See the Supplement.
